# Improving Patients’ Experiences of Ward Round in Psychiatry Wards

**DOI:** 10.1192/bjo.2024.621

**Published:** 2024-08-01

**Authors:** Rifat Binte Radwan, Chiro Islam Mallik

**Affiliations:** Essex Partnership University NHS Foundation Trust, Chelmsford, Essex, United Kingdom

## Abstract

**Aims:**

The aim of the audit is to improve the patients’ experience in the ward round, to ensure all the patients feel included in their ward rounds, to find out if their physical health gets as much attention as their mental health and to establish they receive appropriate feedback of their treatment progress while being inpatient.

By carrying out the audit, our target was to ensure patients are prepared, that service users and carers are involved, clear goals are set, patient confidence is increased, there is direct patient interaction, and the ward round is a positive experience for both the patient and the care team.

**Methods:**

This audit included 34 patients from our acute adult psychiatry mixed unit and perinatal psychiatry unit, who have had at least two ward rounds in the inpatient setting, have the capacity to consent to treatment and have insight to their mental health issues.

Data collection was done by a face to face patient interview with a questionnaire by the junior doctors in the wards. The questionnaire was discussed with the head of patient experience team and clinical audit & improvement facilitator team prior to starting the audit. Confidentiality was maintained and at no point were the patients requested to reveal their identity.

**Results:**

Among the patients admitted during the period of October 2022 to November 2022, 40 patients were chosen randomly. Among them, data was collected from 34 patients according to the inclusion criteria.

Based on the patients’ feedback and experience, 52.9% service users thought the ward round time tables ran by schedule, whereas 11.8% service users thought they did not run by schedule at all.

20.6% service users reported that they were not informed in advance, if there were any change in the ward round time schedule; whereas 41.2% service users were informed of the changed time schedule.

50% service users reported that they received appropriate support prior to the ward round should they need this, on the other hand 8.8% said they did not require any support, therefore refrained from answering the question.

Majority of the service users 94.1% reported that they were happy with the ward round physical environment, the sitting arrangement and the ambience and 88.23% service users reported being reviewed by the consultant at least once a week. None of the service users were found, who was not reviewed by the consultant at least once weekly during their stay in the inpatient wards.

47.1% service user reported that they did not feel confident and comfortable in front of all the professionals present in the ward round.

20.6% service users complained that their physical health was not getting as much attention as their mental health in the psychiatric wards and they were not referred for their physical health needs accordingly.

23.5% of service users had little to moderate understanding on what was being discussed in the ward round meeting, whereas 76.5% understood completely what was being advised in the ward round.

8.8% of the service users did not feel involved in their ward round at all and majority of the service users, 52.9% got regular feedback on the treatment progression and a chance to talk with their named nurse after the ward round.

**Conclusion:**

Ward rounds are the formal meetings where service users come to the professionals with their queries and are informed about their progression on treatment. These meetings should be comfortable and palatable for both sides of the table. Further arrangements are required to improve the settings and make it more service user friendly to get the best outcome from ward rounds.

